# Impact of Protein and Nutritional Support on the Muscular Status of Critically Ill Patients: A Pilot, Perspective, and Exploratory Study

**DOI:** 10.3390/nu17030497

**Published:** 2025-01-29

**Authors:** Marialaura Scarcella, Emidio Scarpellini, Silvia De Rosa, Michele Umbrello, Gian Marco Petroni, Riccardo Monti, Pierfrancesco Fusco, Edoardo De Robertis, Rita Commissari, Ludovico Abenavoli, Jan Tack

**Affiliations:** 1 Anesthesia, Intensive Care and Nutritional Science, Azienda Ospedaliera “Santa Maria”, Via Tristano di Joannuccio, 05100 Terni, Italy; m.scarcella@aospterni.it (M.S.); gm.petroni@aospterni.it (G.M.P.); r.monti@aospterni.it (R.M.); rcommissari@aospterni.it (R.C.); 2 Translationeel Onderzoek van Gastro-Enterologische Aandoeningen (T.A.R.G.I.D.), Gasthuisberg University Hospital, KU Leuven, Herestraat 49, 3000 Leuven, Belgium; jan.tack@med.kuleuven.be; 3 Interdepartmental Center for Biomedical Sciences (CISMed), Trento University, 38121 Trento, Italy; silvia.derosa@unitn.it; 4 Department of Anesthesia and Intensive Care, Santa Chiara Regional Hospital, 38121 Trento, Italy; 5 Anesthesia Department, San Carlo Borromeo Hospital, ASST Santi Paolo e Carlo Polo Universitario, 20019 Milan, Italy; michele.umbrello@unimi.it; 6 Anesthesia Department, Avezzano General Hospital, 67051 Avezzano, Italy; pfusco@asl1abruzzo.it; 7 Anesthesia Department, Perugia University, 06121 Perugia, Italy; edoardo.derobertis@unipg.it; 8 Department of Health Sciences, University “Magna Graecia”, 88100 Catanzaro, Italy; l.abenavoli@unicz.it

**Keywords:** nutritional status, sarcopenia, anabolic resistance, whey proteins, methyl hydroxybutyrate, critically ill patient

## Abstract

Background: Malnutrition and muscle weakness are highly prevalent in critically admitted patients. To overcome sarcopenia and muscle weakness, physical activity and neuromuscular electric stimulation have been introduced with limited efficacy. Thus, several anabolic remedies have been introduced. An adequate increase in protein support according to indirect calorimetry and body composition and methyl hydroxybutyrate (HMB) is emerging. Therefore, we wanted to investigate the impact of HMB-enriched whey formula on the nutritional status, muscle weakness, and clinical course of critically ill patients undergoing nutritional status multimodal assessment and physical rehabilitation. Methods: We consecutively enrolled critically ill adult patients admitted to the intensive care unit (ICU) of “Santa Maria Hospital”, Terni, Italy. All patients underwent preliminary anthropometric, laboratory tests, nutritional (bioimpedance vector analysis and indirect calorimetry), and ultrasound muscle assessment at admission (T0). Laboratory tests monitoring continued throughout the ICU stay. Nutritional and muscle strength assessment was taken weekly throughout the patient’s ICU stay. All patients were enterally administered with a whey protein-enriched formula. Ten days after admission (during the physical rehabilitation period), patients were randomly administered a mixture of essential amino acids and methyl hydroxybutyrate (HMB). Results: We consecutively enrolled 54 ICU patients. At the baseline, survivors (n = 46) were significantly younger than non-survivors. The latter had a worse SAPS II score, nutritional status, and risk, with no significant difference in basal metabolism. Prealbumin values significantly correlated with improved nutritional status and metabolism. Starting from 10 days upon ICU admission, the pennation angle (used as a measure of muscle strength) significantly correlated with the improvement in nutritional status. Whey proteins were well tolerated. Its administration showed a tendency to improve the pennation angle. No specific effect of the mixture containing essential amino acids and methyl hydroxybutyrate was observed. Nutritional status improvement and the rise of basal metabolism were significantly correlated with the extubation time. On the other hand, the reduction in muscle weakness was not significantly correlated with the timing of extubation. Conclusions: Whey protein formula administration can significantly improve nutritional status and basal metabolism in ICU patients. This is reflected in improved muscle strength. Whey protein administration shows a tendency toward a rise in pennation angle. A similar and non-specific trend was observed upon HMB mixture add-one. Further prospective large-scale controlled studies are needed to confirm these promising results.

## 1. Introduction

Patients admitted to the intensive care unit (ICU) by any cause show a relevant incidence of muscular weakness, ranging between 30% and 70% of patients [1]. Besides malnutrition, muscle wasting is the major determinant of muscle weakness [2]. In parallel to malnutrition, ICU weakness can start during the first week of intensive environment admission, with an incidence of up to 20% of patients [3]. Similarly to the larger concept of malnutrition, critical patients’ muscle weakness is significantly associated with longer duration of mechanical ventilation and ICU stay. Finally, weakness is also associated with increased ICU mortality [3,4].

Due to these factors, experts have suggested early nutritional strategies to prevent malnutrition and muscle weakness in patients, mitigating the risks associated with premature initiation of nutrition (specifically, the inhibition of autophagy and reduction in ketogenesis) [5]. These lines of intervention extend beyond treating pathological conditions like sepsis and maintaining glycemic control, and they also encompass early neuromuscular electrical stimulation and physical activity [1]. Unfortunately, these measures did not show a strong efficacy in reducing muscle weakness development [5].

Several anabolic remedies and approaches have been proposed over the last two decades. Among these, whey proteins show more consistent evidence [6], and beta-hydroxy beta-methyl butyrate (HMB) is an emerging approach with peculiar anabolic and, importantly, anti-catabolic properties. In detail, HMB is the metabolite of the essential amino acid leucine [7]. Several randomized-controlled trials (RCTs) have confirmed its efficacy and, importantly, safety in treating sarcopenia and muscle wasting in several conditions such as elderly and cancer [8,9]. In another trial, a mixture of HMB, L-arginine, and L-glutamine was compared to non-essential amino acids in patients with advanced cancer-related cachexia [10]. The treatment group showed a significant increase in fat-free mass. Patients with acquired immunodeficiency syndrome responded similarly to the mixture [11]. However, there is no consistent agreement yet on the efficacy of the HMB add-on used in critical patient nutrition to counteract and reverse muscular weakness/sarcopenia with significant clinical relevance.

Therefore, we wanted to assess the potential effect of daily HMB-enriched whey formula on the nutritional status and muscle weakness of critically ill patients undergoing nutritional status multimodal assessment and physical rehabilitation. The secondary aim was to assess patient outcomes and their clinical course during the ICU stay.

## 2. Materials and Methods

### 2.1. Study Protocol

In this single-center prospective exploratory study, we consecutively enrolled critically ill adult patients admitted at the ICU of “ Santa Maria Hospital”, Terni, Italy between 1 January and 30 July 2023. We respected regional Ethical Committee rules for patients’ enrollment (Ethical Committee CEAS Umbria, Italy, provisional CER N3650/23, March 2023; final approval: CER N3750/23, 30 July 2023). Enrollment was operated upon patients’ or relatives’ informed consent signature and approval.

**Figure 1:**
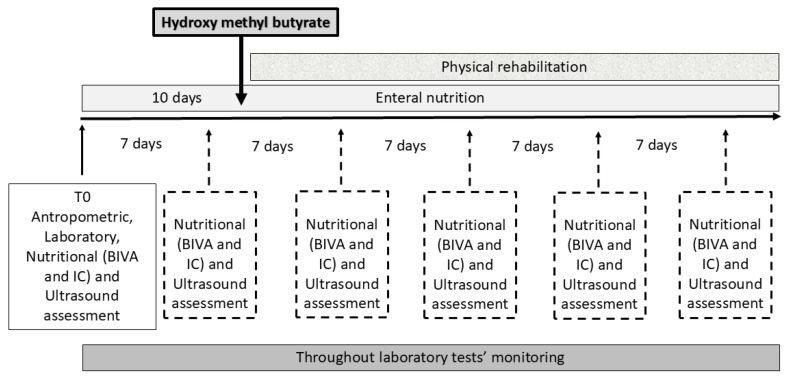
Study design representation. All patients underwent preliminary anthropometric, laboratory tests, nutritional (bioimpedance vector analysis and indirect calorimetry), and ultrasound muscle assessment at admission (namely, T0). Laboratory test monitoring continued throughout the ICU stay. Nutritional and muscle strength assessment was taken weekly throughout the patient’s ICU stay. Upon whey protein formula administration, from day 10 onward, patients were randomly assigned to a mixture of essential amino acids and hydroxy methyl butyrate.

Patients older than 18 years and in need of mechanical ventilation for at least 24 h were included in the study (Figure 1).

The exclusion criteria were pregnancy, artificial nutrition in the previous 15 days upon admission, allergy to the whey protein formula components, major gastro-intestinal surgery, malabsorption syndromes, inflammatory bowel disease, gastro-intestinal motility disorders, acute/chronic pancreatitis, immunodepression (e.g., acquired immunodepression syndrome (HIV)), hematologic disease, and cognitive status impairment.

### 2.2. Nutritional Strategy Scheme

Each patient followed a standardized enteral nutritional (EN) protocol [9], as follows:

- EN was started 24 or 48 h after ICU admission. It has to cover 50% of caloric requirements;
- After at least 48 h, EN had to reach 80–100% of caloric needs;
- The protein target was set at ≧1.3 g/kg/day [12,13];
- The caloric target was set at 15–20 kcal/kg/day;
- It used a high-protein (containing 100% whey proteins) and casein-free formula, low in carbohydrates (CHO), and with a fat/CHO ratio of 50/50. It was rich in Medium Chain Triglycerides (MCTs) and omega-3 fatty acids, with complete vitamins, minerals, and a trace elements profile.

Patients were fed the EN formula also when in the prone position and maintained at least 10% of bed anti-Trendelenburg tilt. The same was applied to patients treated with non-invasive ventilation (NIV) and in the conditions of controlled hypoxemia and permissive hypercapnia.

EN was started at a 10–20 mL/h infusion rate. Its speed of infusion increased by 10 to 20 mL/h every 24 h, based on the gastric tolerability. In the case of 1% propofol infusion, the calories and amount of lipids provided with this drug were included in the daily energy balance. In obese patients, nutritional targets were calculated considering Body Mass Index (BMI) and Ideal Body Weight (IBW).

Calories:
In total, 11–14 kcal/kg current body weight/day, for BMI of 30–50 kg/m^2^;
In total, 22–25 kcal/kg IBW, for BMI > 50 kg/m^2^;
In total, 65–70% of the measured calories.
Protein intake:
In total, 2 g/kg IBW for BMI 30–50 kg/m^2^;
In total, 2.5 g/kg IBW for BMI > 50 kg/m^2^.

The needs for trace elements and vitamin supplementation were evaluated in previously malnourished patients or in cases of partial EN with or without complementary parenteral nutrition (250 mL of 5% glucose solution administered for 5 to 6 h during the night once a day for three days as a loading dose and then twice a week as the maintenance dose, respectively) [11,13].

Starting from day 10 onward, patients were randomly administered a mixture of essential amino acids and HMB (precisely, L-Leucine 1250 mg, Calcium Hydroxy methyl butyrate (HMB) 750 mg, L-Lysine 650 mg, L-Isoleucine 625 mg, L-Valine 625 mg, L-Threonine 350 mg, L-Cysteine 150 mg, L-Histidine 150 mg, L-Phenylalanine 100 mg, L-Methionine 50 mg, L-Tyrosine 30 mg, L-Tryptophan 20 mg, Vitamin D (Cholecalciferol) 10 mcg, vitamin B6 0,15 mg, vitamin B1 0,15 mg. Protein 0,00 g; Carbohydrates 1787 g; fibers 0.124 g, fat 0.065 g) dissolved in water 150 mL b.i.d., coincident with the beginning of physical rehabilitation. The randomization was performed blind according to a computer-assisted randomization model (1:1 ratio).

### 2.3. Indirect Calorimetric Measurements

The resting energy expenditure (mREE) data were obtained through a Q-NRG+^®^ Metabolic Monitor (Baxter, Rome, Italy). At admission, patients were temporarily excluded from indirect calorimetry (IC) assessment in the case of the fraction of inspired oxygen (FiO2) > 70%, hemodynamic instability, positive end-expiratory pressure (PEEP) > 16 mmHg on active veno-venous extracorporeal membrane oxygenation (VV ECMO), or per ICU attending clinical judgment. IC data were recorded at 10- to 30-min intervals. Thus, they met steady-state conditions defined by a variance of volume of oxygen (V02) and volume of carbon dioxide (VC02) by <10% as per published validation data for the Q-NRG device [10]. The data were averaged over weekly intervals for patients with multiple IC measurements.

### 2.4. Bioimpedance Vector Analysis

Bioelectrical impedance analysis (BIA) is a non-invasive tool to assess human body composition (e.g., fat, bone, water, and muscle content). The tool delivers a low-frequency electrical current according to the principle that fluid and cellular structures present different levels of resistance to the current passing through a living system. BIA measurements are resistance (R-Ohms), describing cellular hydration; reactance (Xc—Ohms), describing tissue integrity, and phase angle (PhA—degrees), which is the arc tangent between R and Xc. Thus, BIA evaluates hydration and nutrition in men [11].

Bioelectrical impedance vector analysis (BIVA) assesses body composition in patients with advanced illnesses, such as those admitted to intensive care. In fact, statistical vector analysis of BIA data better describes body composition in this subset of patients [12]. In detail, BIVA uses graphical vectors to analyze BIA data. Thus, impedance (Z) is plotted as a vector from its components R (*X*-axis) and Xc (*Y*-axis), after being standardized by height (H). The RXc graph represents the sex- and race-specific tolerance intervals of a comparative reference population. Tolerance ellipses are plotted on the RXc graph to represent the 50%, 75%, and 95% centiles (i.e., confidence intervals) for the population in the study. This method allows a simultaneous assessment of tissue hydration or soft tissue mass changes independent of regression equations or body weight. For these reasons, BIVA can also be interpreted accurately in critically ill ICU patients at extremes of weight or volume distribution.

### 2.5. Nutritional Risk Scores Assessment

The mNUTRIC score includes five variables: age, APACHE II, SOFA, number of comorbidities, and days from hospital to ICU admission. It was calculated using data from the first 24 h after ICU admission. The mNUTRIC score ranges from zero to nine; a score ≥ 5 indicates a high nutritional risk [13].

### 2.6. Prognostic Score Assessment

The SAPS II score comprises 17 components, including 12 physiological variables, age, admission type, and underlying disease. It estimates mortality rates for different medical conditions [13].

### 2.7. Muscle Pennation Angle Ultrasound Assessment

The pennation angle is the angle of insertion of muscle fibers into the aponeurosis. Therefore, the angle describes muscle architecture. More in detail, the measurement describes muscle strength. In fact, the greater the pennation angle, the more the contractile material is packed within a given volume and the higher the muscle’s capacity to generate force [14]. Thus, muscles with larger pennation angles are thicker because they have greater numbers of sarcomeres in parallel with the direction of the fascicle. These parallel sarcomeres may be lost first, causing loss of the pennation angle as an indication of reduced thickness. In anasarca patients, pennation angle measurement can be biased by intramuscular edema presence. In this case, measurement correction must be considered [15]. The measurement of the rectus femoris pennation angle was conducted with subjects in a supine position using a 10–12 MHz multifrequency linear matrix probe. The probe was positioned perpendicular to the transverse axis of the dominant leg (lower third of the distance between the iliac crest and the upper border of the patella) [16].

### 2.8. Data Collection

All clinical and laboratory data were prospectively collected from the patient’s medical file. General and demographic variables on the day of ICU admission were recorded, and the SAPS II score was calculated to obtain information on the severity of critical illness [15]. All other variables were recorded daily for the entire ICU stay, starting from admission to discharge/death: albumin, total protein, serum prealbumin, inflammation and infection markers (C reactive protein (CRP), white blood cells count and formula, procalcitonin and erythrocyte sedimentation rate), renal and hepatic function indices, and blood gas analysis variables. The main evaluated outcomes were the ICU mortality and the duration of mechanical ventilation (extubation time). All collected data were included in a database warranting the patients’ anonymity and using numbers as identification codes for the subjects.

### 2.9. Statistical Analysis

Statistical analyses were performed using SPSS Software 21 (IBM, New York, NY, USA). Quantitative variables distribution was tested with the Kolmogorov–Smirnov normality test. All data are presented as mean ± standard deviation (SD) or median [interquartile range, IQR] according to the normal or not normal distribution. Parametric (Student’s *t*-test) and non-parametric tests (Mann–Whitney U test) were applied to describe the differences between groups for the variables of interest as appropriate. Analysis of variance was performed to assess variable modifications over time, with correction for multiple tests. Kaplan–Meier survival curves for the comparison of the Hazard Ratio between groups were built for the survival analysis. The alpha level of significance was set at 0.05 [17]. The sample size was calculated to obtain the alpha level of significance with a 1:1 ratio of comparison (https://clincalc.com/stats/samplesize.aspx (accessed on 30 November 2024)).

## 3. Results

We consecutively enrolled 54 ICU patients. The main comorbidities of patients were diabetes (20%, type 2 90%), hyperuricemia (15%), hypertension (40%), chronic ischemic heart disease (22%), chronic obstructive pulmonary disease (COPD) (27%), anxiety (10%), and depression (7%).

The study population had a mean age of 66.1 ± 5.6 years and a BMI of 21.4 kg/m^2^, with a mean SAPS II score of 58.1 ± 9.0 points at the admission.

Patients’ general, anthropometric, and nutritional characteristics are shown in Table 1.

At baseline, survivors were significantly younger than non-survivors. The latter had a worse SAPS II score and nutritional status and risk, evaluated with BIVA and mNUTRIC score, respectively (all *p* < 0.05). Basal metabolism did not differ significantly among survivors and non-survivors (*p* = NS). Deaths occurred at 11 (n = 2), 16 (n = 3), and 23 (n = 3) days upon admission.

According to BIVA measurements, survivor nutritional status was distributed as follows: malnourished (M) (n = 10, 22%, PhA of 3.6°), overweight (O) (n = 11, 24%, PhA of 5.8°), and well-nourished (W) (n = 25, 54%, PhA of 4.8°). The body fat mass of overweight and obese patients was 33%.

Non-survivors were mainly malnourished (50%) and overweight (25%). The latter had a body fat mass of 37%.

The whey protein formula was well tolerated. In detail, all patients had a mean daily caloric intake ranging from 18 to 25 kcal/kg/day and a mean daily protein intake of 1.3 g protein/kg/day.

All surviving patients reached a target of protein intake of 1.4 g/kg/day. The median achievement time was 3.6 ± 0.2 days (range 2.8–6.1).

Three patients among the non-survivors did not reach the protein intake target (target time of 3.6 ± 0.5 days). Interestingly, the severity of disease (according to a higher SAPS II) was associated with a longer time to reach the protein target (r = 0.33).

**Figure 2:**
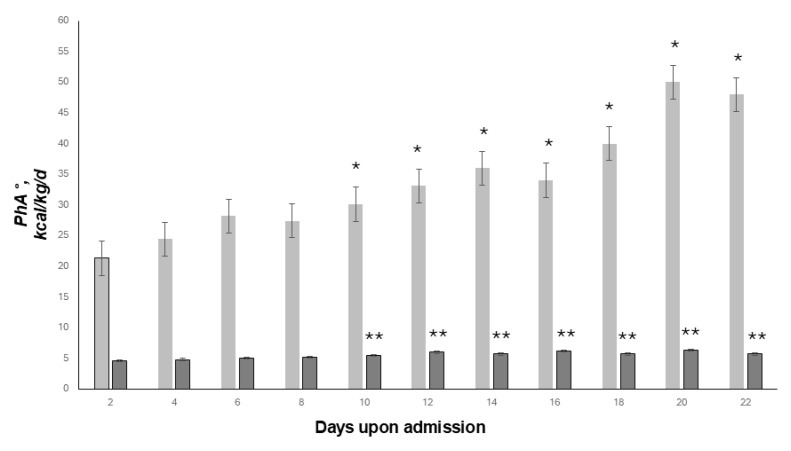
Improvement in nutritional status and basal metabolism among survivor patients. Starting from day 10 onward, nutritional status (dark grey, PhA °) and basal metabolism (light grey, kcal/kg/day) significantly improved vs. baseline in survivor patients only (ANOVA, both (* and **) *p* < 0.05).

During the follow-up period, survivors showed a significant improvement in nutritional status and rise of basal metabolism, assessed through BIVA and indirect calorimetry, respectively, and expressed as PhA and mREE (ANOVA, both, *p* < 0.05) (Figure 2).

Non-survivor patients did not show a significant improvement in nutritional status and basal metabolism did not rise significantly (ANOVA, both, *p* = NS).

Potential cofounding factors (e.g., different sedatives dosage) were ruled out.

**Figure 3:**
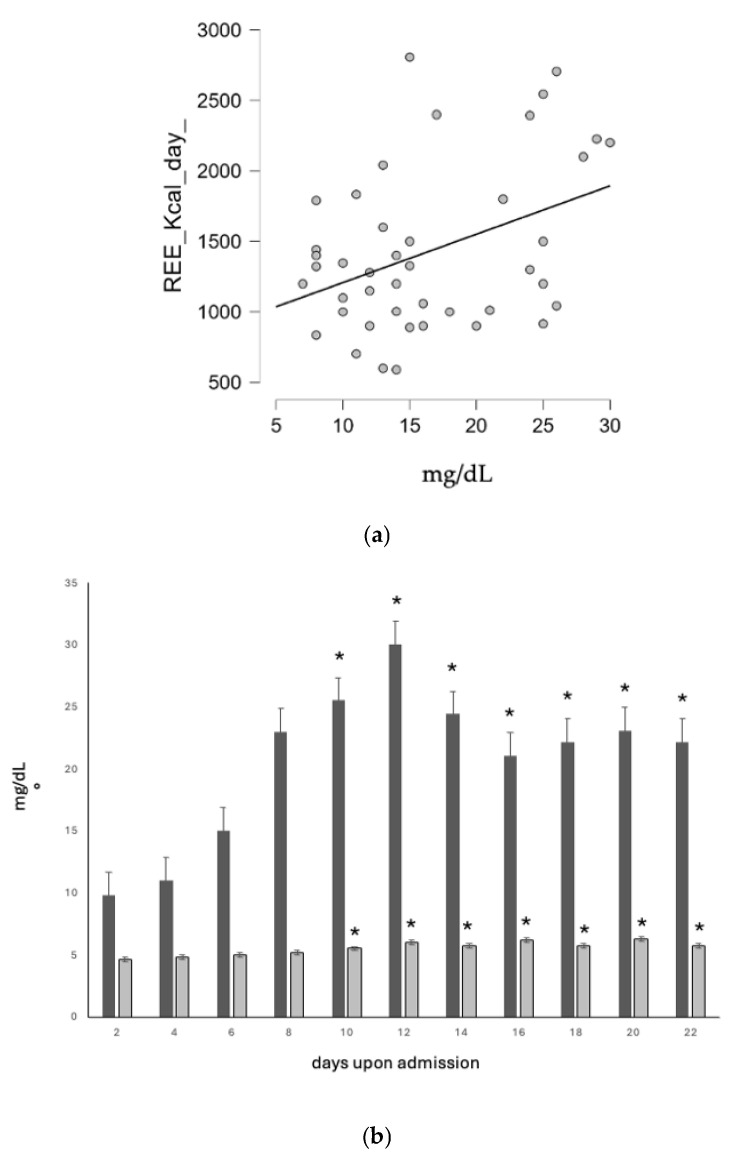
(**a**). Correlation between basal metabolism and prealbumin values (r = 0.34). Prealbumin values significantly rose and correlated with those of basal metabolism, assessed with IC. (**b**). Nutritional status (light grey, PhA °) and prealbumin values (dark grey, mg/dL). Prealbumin values significantly rose and correlated with those of nutritional status, assessed with BIVA (*, r = 0.37).

Moreover, starting from day 10 onward, the rise of prealbumin values, used as a hybrid prognostic and nutritional status index, was significantly correlated with the improvement in basal metabolism and in nutritional status (r = 0.34 and r = 0.37, respectively) (Figure 3a and Figure 3b, respectively).

**Figure 4:**
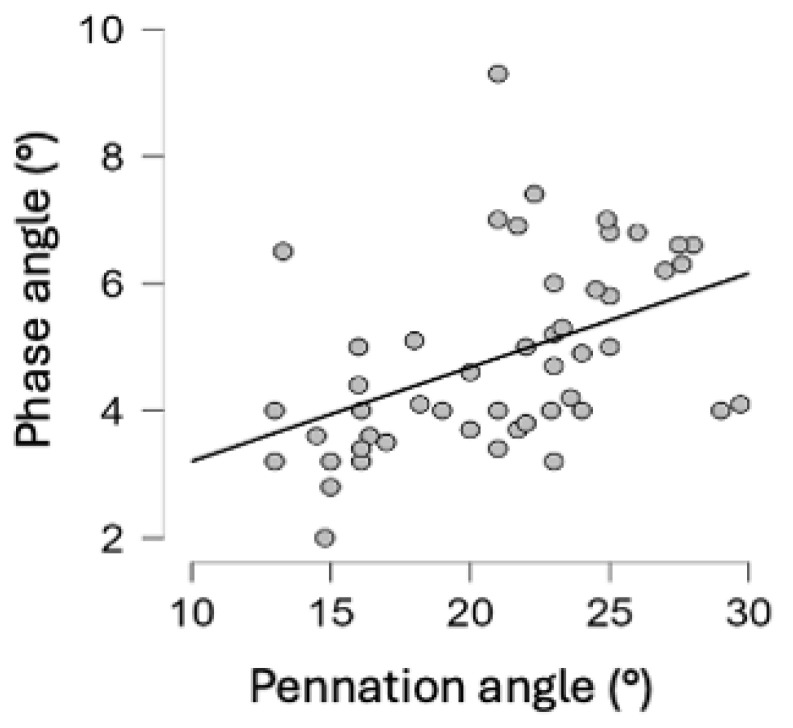
Correlation between nutritional status and pennation angle (r = 0.371).

Muscle strength, measured by the pennation angle of the rectus femoris muscle, was significantly correlated with the improvement in nutritional status, expressed as PhA (r = 0.371, Figure 4). The correlation was statistically significant starting from 10 days upon admission.

**Figure 5:**
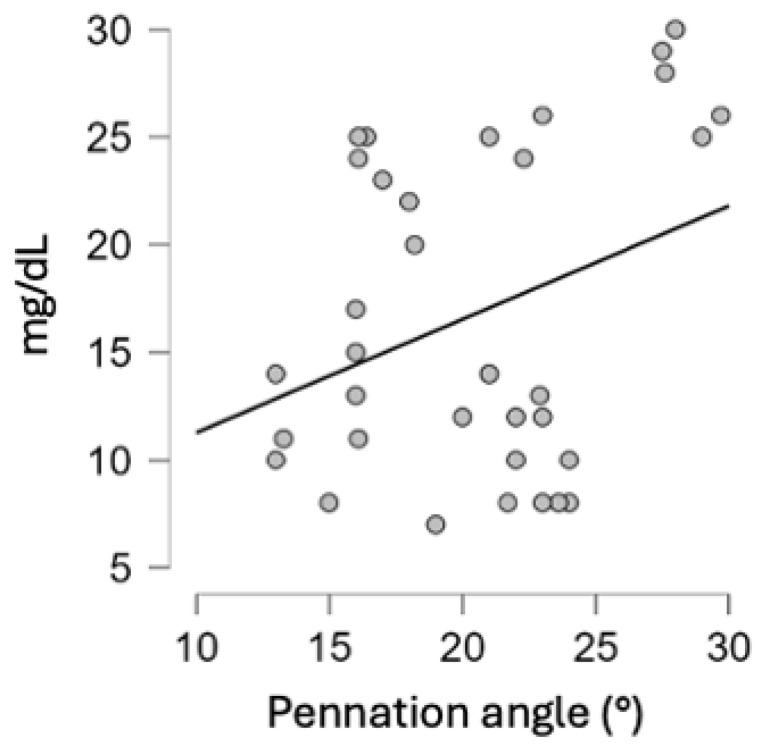
Correlation between prealbumin values and the pennation angle (r = 0.354).

Similarly, the rise of prealbumin values was significantly correlated with the reduction in muscle weakness, represented by a significant increase in pennation angle (r = 0.354, Figure 5). Importantly, the correlation was statistically significant, starting from 10 days upon admission.

**Figure 6:**
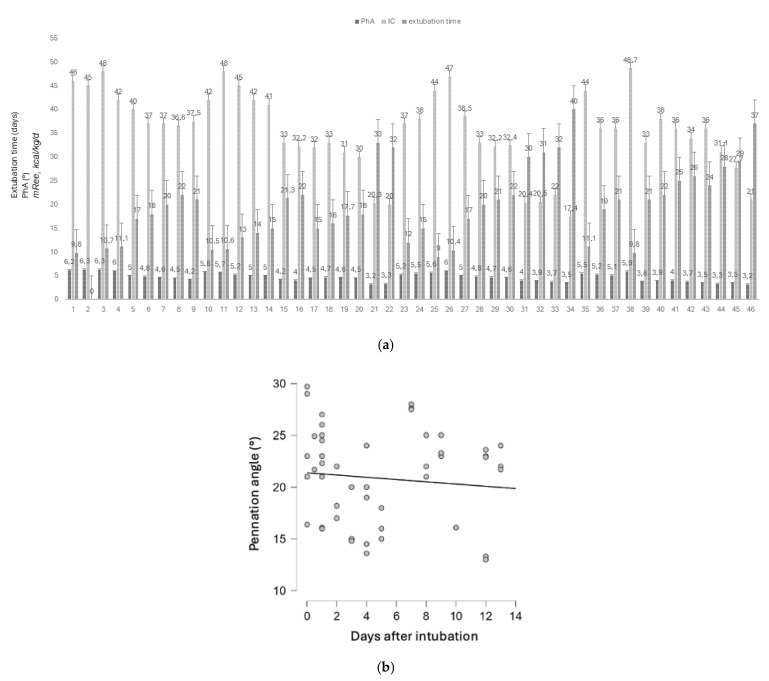
(**a**). Correlation between nutritional status, basal metabolism, and extubation time. Survivor patients showed a significant correlation between improved nutritional status (PhA °), basal metabolism (IC, mREE, kcal/kg/d), and extubation time, r = 0.38 and 0.332. For each patient, the last PhA and IC value available before the extubation are represented. (**b**). Muscle strength (measured with the pennation angle) and extubation time (days). The improvement in the pennation angle did not correlate with the extubation time, r = NS.

Nutritional status improvement and a rise of basal metabolism were significantly correlated with the extubation time (r = 0.38 and 0.332, respectively) (Figure 6a).

Interestingly, the reduction in muscle weakness was not significantly correlated with the timing of extubation during the ICU stay (r= −0.09) (Figure 6b).

The patients with shorter mechanical ventilation and successful extubation showed a tendency to have higher serum prealbumin levels vs. non-survivors

Hydroxy methyl butyrate was well tolerated (5 patients reported mild flatulence), and all patients in whom it was administered (n = 25) completed the supplementation during the physical rehabilitation period. There was no difference in treatment compliance among survivors and non-survivors (n = 21 vs. 4). The mortality distribution within this group of patients was 1, 1, and 2 deaths at 11, 16, and 23 days upon admission, respectively. The patients non-supplemented with HMB (n = 29) recorded a total of 5 deaths: 1 occurred at 11, 2 at 16, and 2 at 23 days upon admission, respectively. There was no statistical difference in mortality rate or distribution between the two groups (both, *p* = NS).

Importantly, there was no significant difference in the EN whey formula composition and administration protocol among the HMB-supplemented group (n = 25) and non-HMB supplemented group (n = 29). In detail, the reached target of protein intake was of 1.4 vs. 1.41, 1.38 vs. 1.39, and 1.4 vs. 1.38 g/kg/day (starting from 10 upon ICU admission, 20 and 30 days later, respectively, all *p* = NS). The caloric targets reached were 25–40 vs. 24–39, 29–47 vs. 29–46, and 32–43 vs. 31–43 kcal/kg/day (starting from ten upon ICU admission, 20 and 30 days later, respectively, all *p* = NS). The administration speed of the formula was not significantly different among groups: 35 vs. 37, 42 vs. 42, and 48 vs. 47.5 mL/h (starting from ten upon ICU admission, 20 and 30 days later, respectively, all *p* = NS).

**Figure 7:**
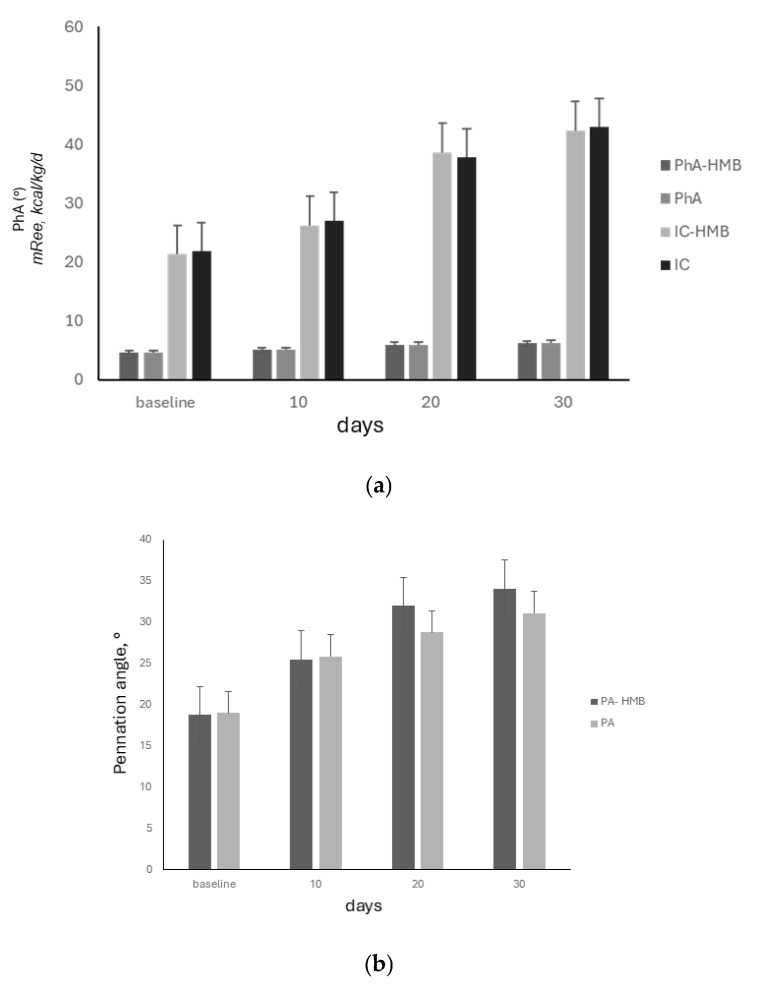
(**a**). HMB administration, nutritional status, and basal metabolism. During the physical rehabilitation period, HMB mixture administration did not significantly affect the improvement in nutritional status and basal metabolism of survivors (ANOVA, ten days upon ICU admission vs. 20 and 30 days, all *p* = NS). Legend: PhA-HMB, PhA, phase angle in HMB-administered and non-administered patients, respectively. IC-HMB and IC, indirect calorimetry in HMB-administered and non-administered patients, respectively. (**b**). HMB administration and pennation angle delta. Survivor patients administered with the HMB mixture showed the tendency to have higher pennation angle values at 20 and 30 days vs. ten days upon ICU admission, ANOVA, *p* = 0.08, *p* = 0.09, respectively.

Interestingly, HMB supplementation did not significantly affect nutritional status and basal metabolism (ANOVA, both *p* = NS) (Figure 7a). In detail, there was no significant difference among survivors and non-survivors in the HMB-supplemented group (survivors, n = 21 vs. non-survivors, n = 4: 10, 20 and 30 days upon ICU admission: PhA: 5.01 vs. 4.9, 5.88 vs. 5.70 and 6.12 vs. 5.90°; ICC: 26 vs. 25.3, 39 vs. 37.1, 43 vs. 41.4 kcal/kg/day). Thus, death occurrence did not significantly affect nutritional status and basal metabolism in the HMB-supplemented group. Similar results were observed in the non-HMB supplemented group.

HMB administration showed a tendency to affect the pennation angle delta, without reaching statistical significance (ANOVA, *p* = 0.08, *p* = 0.09, ten days upon ICU admission vs. 20 and 30 days, respectively) (Figure 7b). There was no statistically significant difference among survivors and non-survivors (n = 21 and n = 4, 25 vs. 23, 32 vs. 30 and 35 vs. 32.4°, at 10, 20 and 30 days upon ICU admission, respectively, all *p* = NS).

Similarly, in the non-HMB administered group, there was no statistically significant difference for pennation angle delta among survivors and non-survivors (n = 25 and n = 5, 24.4 vs. 23.7, 32.2 vs. 29.2, and 35.7 vs. 32.7°, at 10, 20, and 30 days upon ICU admission, respectively, all *p* = NS).

Finally, death occurrence was associated with worse SAPS II and mNUTRIC scores, irrespective of HMB supplementation (HMB-supplemented group (n = 25): SAPS II, 50 ± 4.2 vs. 70.1 ± 4.8, 48.1 ± 4.0 vs. 71.2 ± 4.1, and 45 ± 4.6 vs. 73.3 ± 5.1, survivors vs. non-survivors, values at 10, 20, and 30 days upon admission, all *p* < 0.05; mNUTRIC, 3 vs. 7, 3 vs. 8, 2 vs. 8, survivors vs. non-survivors, values at 10, 20, and 30 days upon admission, all *p* < 0.05; non-HMB-supplemented group (n = 29): SAPS II, 49.9 ± 4.1 vs. 70.1 ± 3.9, 49.1 ± 4.0 vs. 72.6 ± 5.2, and 43.2 ± 4.7 vs. 74.5 ± 5.0 survivors vs. non-survivors, values at 10, 20, and 30 days upon admission, all *p* < 0.05; mNUTRIC, 3 vs. 8, 2 vs. 8, 2 vs. 8, survivors vs. non-survivors, values at 10, 20, and 30 days upon admission, all *p* < 0.05).

## 4. Discussion

In this prospective single-center exploratory study, we have shown that the administration of whey protein formula is associated with improved basal metabolism and nutritional status. Prealbumin levels significantly correlated with both improved basal metabolism and nutritional status. Prealbumin values and related improvement in nutritional status, assessed with PhA, significantly correlated with improved muscle strength, assessed by ultrasound of the rectus femoris pennation angle. The improved nutritional status and basal metabolism significantly correlated with a shorter extubation time. On the other hand, the improved pennation angle did not correlate with an earlier extubation time.

Finally, the randomized add-on of HMB enriched mixture showed a tendency toward an improved pennation angle during the physical rehabilitation period. No effect on nutritional status, basal metabolism, and mortality was observed.

An adequate nutritional risk assessment and nutritional approach with a formula rich in proteins have shown consistent evidence of efficacy in critically ill patients. In detail and historically, high-protein enteral formula studies have demonstrated the capability to significantly reduce the mortality and morbidity of ICU patients compared to placebo [18]. Conversely, patients intolerant to enteral nutrition formula (e.g., for gastrointestinal intolerance) present with poorer nutrition and clinical outcomes (precisely, increased ICU stay and mortality) [19]. Whey protein-rich enteral formula has the proteins with the highest biological value due to their high concentration of essential amino acids. This translates into better and faster digestion and metabolism compared to caseinates. Therefore, they provide better amino acid uptake for muscle anabolism, especially in ICU patients presenting with sarcopenia. In addition, whey proteins have a high cysteine content and are the best source for glutathione peroxidase functioning, resulting in increased clearance of free radicals. This process is crucial and is curative for the reversal of “inflamed“ ICU patients of any cause (e.g., trauma, postoperative, sepsis, multiorgan failure) [20]. In accordance, a previous report from our research group has clearly shown how whey protein-rich enteral formula can significantly improve nutritional status in COVID-19 patients admitted to the ICU and shorten their extubation time [21].

Prealbumin is a tetrameric protein and accounts for 15% of albumin serum levels. Because of its short half-life of two days, it was first considered to follow up rapid changes in the metabolic status of critically ill patients [22]. However, prealbumin is also a negative acute phase reactant and inflammatory cytokines reduce its hepatic synthesis. Thus, its serum concentrations may decrease despite sufficient nutrition supplementation and in the absence of malnutrition [23]. Indeed, the real biodynamic of prealbumin is represented by fluctuations according to those of catabolism upon acute stressors [24]. Therefore, serum prealbumin levels can be considered a hybrid catabolism–inflammation prognostic index in critically ill patients [25].

Results from the study confirm the significant correlation between an increase in prealbumin levels and improved nutritional status and depressed basal metabolism of ICU patients. This was followed by a significant reduction in inflammatory markers.

Results from our investigation confirmed a significant association between improved PhA and IC values and earlier extubation time. This finding is in strong agreement with the available literature [26,27]. Rising prealbumin values did not correlate with earlier ventilation weaning times. These data reflect the hybrid nutritional/inflammatory nature of prealbumin used as a biomarker, not allowing its linear use as exclusive malnutrition or exclusive inflammatory index [28].

Indeed, our study retrieved a significant correlation between prealbumin values and the pennation angle used as a validated tool for muscle strength. This finding can be explained by prealbumin serum levels reflecting both improved nutritional status and muscle anabolism [29]. However, in this case, prealbumin should be considered an index of sarcopenia. In fact, some reports from the literature describe its inverse correlation with sarcopenia diagnostics criteria in non-critical patients [28]. This inverse correlation also has historical evidence [29].

In our study, improved muscle strength did not correlate with an earlier extubation time and reduced rate and distribution of mortality. The findings on extubation time and mortality are not in agreement with the literature. In fact, there are encouraging data supporting a significant correlation between improved muscle strength and earlier weaning from ventilation in critically ill patients [30]. In our study, the lack of correlation can be explained by the tendency toward improvement in the pennation angle obtained upon HMB mixture administration during the physical rehabilitation period. However, this rise did not reach statistical significance. Indeed, we used an HMB-containing mixture with an HMB amount lower than 3 g per day, the maximum tolerated dosage in previous investigations but the highest allowed dose according to the product SmPC [31]. Furthermore, the difference between the present and other studies can be also explained by the timing of the HMB administration, which varies from one trial to another one (e.g., within 2 days upon ICU admission vs. 10 days after) [32,33,34]. In detail, in the study by Hsieh et al., 34 mechanically ventilated COPD patients were randomly assigned to HMB (3 g/day) (n = 18) or placebo (n = 16) for 7 days. Interestingly, white blood cell count, CRP, and creatinine were significantly lower, while cholesterol and total protein were significantly higher after HMB supplementation. Importantly, 10 subjects (55.6%) in the HMB group and 4 subjects (25.0%) in the control group, respectively, had improved pulmonary ventilated function [33]. Oppositely, in the study by Kuhls et al., 100 adult trauma patients received HMB or a combination of HMB, arginine, and glutamine or placebo for 28 days. Surprisingly, HMB alone improved nitrogen balance and not because of lowered muscle protein turnover [32]. In line with these findings, in the more recent RCT by Nakamura et al., 164 ICU patients were screened, and 88 severely ill patients were included and randomly assigned to the control (n = 43) and to the HMB (n = 45) groups, respectively. From day two upon admission, the treatment group was supplemented with 3 g of HMB, 14 g of arginine, and 14 g of glutamine daily, together with a standard nutrition scheme. Early rehabilitation with electrical muscle stimulation was started from day 2 in both groups. HMB failed to significantly prevent femoral muscle volume loss [34]. In the study by Viana et al., 30 ICU patients on mechanical ventilation and with a functional gastrointestinal tract were randomized to HMB (3 g/day) or placebo (maltodextrin) from day four on for one month. The magnitude of reduction in skeletal muscle area (SMA) of the quadriceps femoris was measured by ultrasound at days 4 and 15. Moreover, body composition, change in protein metabolism, and global health were assessed at 60 days upon admission. Interestingly, the loss of total SMA was 11% between days 4 and 15 (*p* < 0.05). There was no difference between groups. In the HMB group, protein breakdown and the production of several amino acids were significantly reduced. PhA increased more in the HMB-treated arm vs. placebo (*p* = 0.0247) [35].

Thus, the timing of HMB administration is a crucial point because it can significantly affect plasma and muscle concentration and retrieve different nutritional absorptive patients’ conditions [36]. For example, early administration can find a hypo trophic muscle compared to administration later during the ICU stay when patients can initiate physical and/or electrical muscle stimulation [36]. In detail, HMB administration seems to reach the peak anabolic effect on patients’ muscles upon starting physical activity [36].

Our study has several limitations: it is a single-center assessment, potentially limiting the significance of results; the sample size is relatively small, although sufficient to compare treatment and control groups according to a 1:1 ratio; and the cause of ICU admission of our patients is variable and this can affect the homogeneity of results, also in comparison with previous reports on the literature focusing on specific ICU subpopulations [36]. In line with this, other factors not related to the nutritional status and interventions could affect the results. This does not allow us to draw definitive conclusions; the simultaneous administration of a standard whey protein-rich formula and HMB-containing mixture (treatment arm) does not allow us to distinguish the relative impact of these nutrients on muscle strength and on the anabolic response in the ICU framework. The future use of a placebo arm would help to distinguish these effects. In addition, the dosage of the HMB-rich mixture is below the agreed maximum amount used in previous studies, although it reflects those used in current clinical practice; the follow-up does not permit a long-term assessment of the impact of nutritional interventions on recovery and clinical outcomes in these patients; and prealbumin has the bi-dimensional nature of being a nutritional status marker and a negative acute phase reactant. Thus, it cannot accurately describe the impact of inflammation on its levels and can explain the risk of misuse as a pure nutritional status biomarker. For these reasons, we have used it as a prognostic index.

In conclusion, this prospective exploratory single-center study showed that whey protein formula can improve nutritional status and increase reduced basal metabolism of ICU patients. This is reflected in earlier weaning from ventilation. During the physical rehabilitation period, HMB mixture administration was followed by the tendency toward increased muscle anabolism. Indeed, this did not reach statistical significance. No significant impact on nutritional status, basal metabolism, or mortality was observed.

Furthermore, larger randomized and controlled studies are needed to confirm the results of the present investigation. In detail, interventional studies are needed to compare the separate and/or additive weight of whey protein-rich formulas and HMB administration on nutritional status and basal metabolism, on the one hand, and muscle strength, on the other hand, in critical patients during their physical rehabilitation phase. In addition, it is crucial to assess the correct timing of HMB administration to reach its peak concentration within muscles and its optimal usage by myocytes, with the expected anabolic output.

From a clinical point of view, the use of whey protein formula can achieve a significant improvement in nutritional status and early ventilation weaning. The HMB add-on could help to overcome the anabolic resistance of critically ill patients, with positive prognostic perspectives in terms of physical rehabilitation.

## 5. Conclusions

The administration of whey protein formula is associated with improved basal metabolism and nutritional status. The latter were associated with significant reductions in prealbumin levels.

Importantly, improvement in nutritional status and the consensual reduction in prealbumin values significantly correlated with improved muscle strength.

From a prognostic point of view, the improved nutritional status and basal metabolism significantly correlated with earlier ventilation weaning. However, the increased muscle anabolism did not correlate with earlier extubation time.

Finally, the randomized add-on of HMB enriched mixture showed a tendency toward improved muscle strength during the physical rehabilitation period, without reaching statistical significance. No significant impact on nutritional status, basal metabolism, or mortality was observed.

The relatively yet adequate small sample size for an exploratory study, the variegate profile of causes for patients’ ICU admission, the short-term follow-up, the mixed nutritional and inflammatory nature of prealbumin as a biomarker, and the consensual whey protein formula and HMB administration can be considered limitations of the study. Altogether, they do not allow us to drive definitive conclusions.

Thus, larger interventional randomized and controlled studies are needed to confirm these promising results.

## Figures and Tables

**Table 1:** Patients’ characteristics at enrollment and outcome.

	Patients (n)	Age (years)	SAPS II	IC (mRee, kcal/kg/d)	BIVA (PhA, °)	mNUTRIC Score
**All patients**	54	66.1 ± 5.6	58.1 ± 9.0	21.3 ± 1.0	4.6	3
**Non survivors**	8	75.7 ± 8.7 *	70.2 ± 5.9 **	25.4 ± 1.2	3.8	7
**Survivors**	46	64.2 ± 6.3	50.4 ± 4.7	22.1 ± 1.1	4.7 ***	3 ****
***p*-value**		<0.05	<0.05	NS	<0.05	<0.05

Table legend: *, **: both *p* < 0.05; NS: non-significant; IC: indirect calorimetry; BIVA: bioimpedance vector analysis. Non-survivors were significantly older than survivors and had a worse SAPS II score (severity of illness) (* and **, both *p* < 0.05). Indirect calorimetry data showed no statistical difference in the metabolism of survivors vs. non-survivors (*p* = NS); on the other hand, survivors showed a higher PhA vs. non-survivors (*** *p* < 0.05). In agreement with PhA values, those of the mNUTRIC score were significantly higher in the group of non-survivors vs. survivors, indicating a higher risk of malnutrition (**** *p* < 0.05).

## Data Availability

All the data reviewed in the manuscript can be retrieved from the main medical databases (e.g., PubMed and Medline) and on the websites of the most important nutrition and intensive care international meetings (e.g., ASPEN, ESPEN, ESICM, WICC).
